# Automated contrast-to-noise ratio analysis in chest CT: validation of an open-source segmentation approach

**DOI:** 10.1186/s13244-026-02263-y

**Published:** 2026-04-07

**Authors:** Nikolas Beck, Giulia Baldini, Luca Salhöfer, René Hosch, Sebastian Zensen, Marcel Opitz, Denise Bos, Jannis Straus, Michael Forsting, Felix Nensa, Lale Umutlu, Johannes Haubold, Mathias Holtkamp

**Affiliations:** 1 Institute of Diagnostic and Interventional Radiology and Neuroradiology, University Hospital Essen, Essen, Germany; 2 Institute for Artificial Intelligence in Medicine, University Hospital Essen, Essen, Germany; 3 Institute of Diagnostic and Interventional Radiology, University Hospital Zurich, Zurich, Switzerland

**Keywords:** Artificial intelligence, Tomography (X-ray computed), Image interpretation (computer-assisted), Quality assurance (health care), Body and organ analysis

## Abstract

**Objectives:**

This study aimed to evaluate the feasibility and accuracy of automated contrast-to-noise ratio (CNR) analysis in chest CT using the open-source body and organ analysis (BOA) framework and to validate segmentation modifications for reproducible image-quality assessment.

**Materials and methods:**

This retrospective study analyzed 100 contrast-enhanced chest CTs (mean age 60.2 ± 15 years; 40% female; 50 CTA, 50 CTPA) and validated the approach in an external cancer imaging archive (TCIA) cohort (*n* = 100). Automated BOA segmentations of the aorta, pulmonary trunk, and paraspinal muscles were modified by fat subtraction and binary erosion and compared with manual measurements from three radiologists. Agreement was assessed using statistical testing, Bland–Altman analysis, and intraclass correlation coefficients (ICC).

**Results:**

Unmodified BOA segmentations yielded significantly lower CNRs than manual measurements (all *p* < 0.01, mean difference up to 6.3). Fat subtraction and binary erosion progressively reduced deviations, with the optimized variant (m_erode6 combined with a_erode6 or p_erode6) showing no significant differences from radiologists (*p* ≥ 0.35). In the external TCIA validation cohort (*n* = 100), agreement was excellent (ICC 0.89–0.93), and Bland–Altman analysis demonstrated minimal bias (Aorta: 0.16 [limits of agreement (LoA) –3.47 to 3.80]; PT: 0.42 [LoA –4.03 to 4.87]).

**Conclusions:**

A minimally modified open-source segmentation framework enables fully automated, reproducible CNR assessment in chest CT, achieving expert-level agreement, including robust performance in external validation. This scalable alternative to manual region-of-interest (ROI) measurement streamlines image-quality assessment, facilitates protocol optimization, and provides standardized metrics ready for integration into AI workflows.

**Critical relevance statement:**

This study provides a validated, fully automated method for quantitative CT image quality assessment, reducing observer dependence and enabling consistent evaluation across scanners, protocols, and institutions, thereby supporting reproducible image quality metrics in clinical routine.

**Key Points:**

- Automated CNR assessment enables objective and reproducible evaluation of image quality in CTA and CTPA.
- Adjustments of the segmentation strategy can substantially improve the accuracy of automated measurements.
- The fully automated approach provides a foundation for standardized and scalable CT image quality analysis in research and clinical practice.

**Graphical Abstract:**

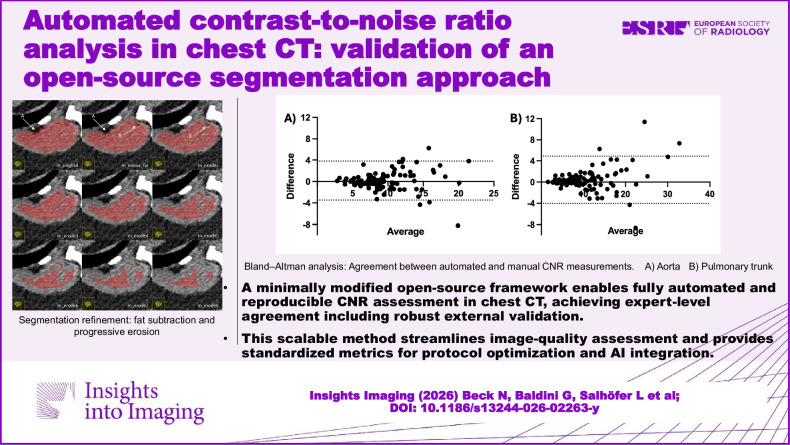

## Introduction

Computed tomography (CT) is a key imaging modality in modern medicine [1]. The quality of CT images is a fundamental prerequisite for diagnostic accuracy, therapy planning [2], and increasingly also for data-driven applications such as radiomics and artificial intelligence (AI) [3–8]. Adequate image quality ensures reliable lesion detection and accurate quantification, particularly in thoracic imaging, where motion susceptibility poses challenges [9–12].

While subjective assessment by radiologists remains the clinical standard for evaluating image quality, it is inherently limited by inter- and intra-observer variability [13]. Moreover, qualitative ratings are not well-suited for large-scale studies, protocol optimization, or AI training pipelines, all of which require reproducible and objective metrics.

Among available objective measures, the contrast-to-noise ratio (CNR) is widely used due to its simplicity and clinical interpretability [2]. However, its routine use is constrained by the need for manual region-of-interest (ROI) placement, which is both time-consuming and observer-dependent [13]. This has led to the development of automated approaches capable of extracting CNR values in a standardized and reproducible manner [14, 15].

Recent advancements in automated image analysis have opened new frontiers in objective CT quality assessment, particularly through tools like the body and organ analysis (BOA) [16]. The BOA framework integrates two open-source segmentation algorithms, TotalSegmentator [17] and Body Composition Analysis (BCA) [18], into clinical workflows. It enables automated segmentation of multiple thoracic structures and facilitates large-scale CNR assessments with minimal manual input. Although initially developed for body composition analysis, its potential for evaluating image quality has not been systematically explored.

Based on the automatically generated segmentations, selective CNR assessment of contrast-enhanced target structures and image noise becomes feasible. However, in manual practice, ROIs are typically placed in the contrast-enhanced lumen, whereas the algorithms segment the entire structure, including the vessel wall. This discrepancy raises concerns regarding the accuracy and reliability of BOA-derived measurements compared to the manual gold standard.

This study aims to evaluate the feasibility and accuracy of BOA-based automated CNR analysis in chest CT. We compare BOA-derived values with expert manual measurements and investigate the impact of various segmentation modifications. Our goal is to establish a reproducible, scalable method for objective CT image quality assessment that supports both clinical and AI-driven applications.

## Materials and methods

### **Ethics statement**

This study was conducted in compliance with the guidelines of the Institutional Review Board of the University Hospital Essen (approval number 24-11698-BO). Due to the retrospective nature of the study, the requirement of written informed consent was waived by the Institutional Review Board. The data were completely anonymized before being included in the study.

### CT and patient cohort

We randomly included 10 studies per CT scanner for each protocol type, computed tomography angiography (CTA) and computed tomography pulmonary angiography (CTPA), resulting in a total of 100 chest CT scans acquired between January 1 and December 31, 2022. Scans were obtained using five different CT scanners (SOMATOM Definition AS, SOMATOM Flash, SOMATOM Edge, and two SOMATOM Force systems; Siemens Healthineers). Mean age was 60.2 ± 15 years (range: 20–85 years; 40% female). Contrast agent (iopromide 300; Ultravist 300, Bayer Vital GmbH) was administered at approximately 1.0–1.25 mL/kg body weight, with an injection rate ranging from 1.9–4.0 mL/s. Bolus tracking with an ROI placed in the aorta for CTA or the pulmonary trunk (PT) for CTPA was used to trigger the appropriate arterial phase. Transverse images were reconstructed using a soft tissue kernel with a slice thickness and increment of 1.0 mm.

### BOA

BOA is an open-source CT segmentation tool based on an nnU-Net architecture [16, 19, 20]. It can be integrated into clinical workflows as a DICOM node and automatically segments the human body with a mean voxel-wise body coverage of 93% ± 2% [16]. By combining TotalSegmentator [17] and BCA [18], BOA enables accurate segmentation of various anatomical structures in medical images, including bones, muscles, organs, and blood vessels, and provides quantitative metrics such as minimum, maximum, and mean Hounsfield units (HU), standard deviation (SD), and volume. In a previous validation study, BOA achieved a mean Sørensen–Dice coefficient of 0.94 across all segmented structures [16]. Recently, an MRI-based extension (BOA-MR) was introduced, enabling fully automated body composition analysis from T2-weighted MRI sequences using nnU-Net models and thereby expanding the clinical scope of the framework [21].

### Examiners

For each CT study, three radiologists with 3, 4, and 5 years of experience in chest imaging (hereafter referred to as Examiners 1–3) were tasked with placing ROIs in the ascending aorta, descending aorta, PT, and autochthonous back muscles (hereafter referred to as muscle), all at the level of the PT. The examiners were instructed to place the ROIs centrally within each structure, maintaining a distance from vessel walls and from adjacent fat tissue surrounding or intersecting the muscle. For each ROI, the following metrics were recorded: minimum, maximum, and mean HU, SD, and area. The aorta was assessed using two ROIs, one in the ascending and one in the descending aorta, with the mean of both used for subsequent analysis. All manual ROI placements were performed using Centricity Universal Viewer 7.0 (GE Healthcare, Chicago, IL, USA).

### Image quality assessment

CNRs were calculated for the aorta and the PT (CNR_aorta_ and CNR_PT_). CNR_aorta_ was defined as the ratio of the difference between the mean HU of the aorta and the muscle to the SD of the muscle. Similarly, CNR_PT_ was calculated as the ratio of the difference between the mean HU of the PT and the muscle to the SD of the muscle.

$${{\rm{CNR}}}{{\rm{aorta}}}=\frac{{{\rm{mean}}}\; {{\rm{HU}}}\; {{\rm{of}}}\; {{\rm{aorta}}}-{{\rm{mean}}}\; {{\rm{HU}}}\; {{\rm{of}}}\; {{\rm{muscle}}}}{{{\rm{SD}}}\; {{\rm{of}}}\; {{\rm{muscle}}}}$$

$${{\rm{CNR}}}{{\rm{PT}}}=\frac{{{\rm{mean}}}\; {{\rm{HU}}}\; {{\rm{of}}}\; {{\rm{PT}}}-{{\rm{mean}}}\; {{\rm{HU}}}\; {{\rm{of}}}\; {{\rm{muscle}}}}{{{\rm{SD}}}\; {{\rm{of}}}\; {{\rm{muscle}}}}$$

### Statistical analysis

All statistical analyses were performed using GraphPad Prism 10.6.1 (GraphPad Software, San Diego, CA, USA). Sample size considerations were conducted to verify that the cohort was sufficiently powered to detect clinically relevant differences between automated and manual CNR measurements. Based on a paired design reflecting repeated measurements within the same examinations (standardized effect size dz = 0.6, α = 0.05, two-tailed, and Δ = 1.0 CNR unit), a minimum sample size of *n* = 24 was required to achieve 80% power. The final internal dataset (*n* = 100 examinations) therefore exceeded this threshold markedly, resulting in an achieved power of approximately 0.98. As some groups exhibited non-normal distribution based on the Kolmogorov–Smirnov test, the non-parametric Friedman test with repeated measures and Dunn’s post hoc test were applied to assess inter-rater variability among the three examiners and between each examiner and BOA. To quantify systematic differences, the mean deviation of BOA-derived CNRs (including all modifications) from the mean CNRs obtained by the examiners was calculated (hereafter referred to as the mean BOA-examiners difference). A *p*-value < 0.05 was considered statistically significant. Descriptive statistics were also obtained for the individual parameters used to calculate CNR.

Agreement between automated and manual CNR measurements was further evaluated using intraclass correlation coefficients (ICC; two-way random effects, absolute agreement, single measures) and Bland–Altman analysis. Bland–Altman plots used the readers’ mean as the primary reference; bias, SD of the differences, and 95% limits of agreement (LoA) were reported. Proportional bias was tested by linear regression of differences on means. Outliers were reported, and sensitivity analyses excluding extreme values were performed. All comparisons between BOA and human readers were performed within the same examinations, ensuring that acquisition protocol factors (e.g., contrast dose, injection rate, CTA vs CTPA) equally affected both approaches and did not bias the agreement analyses.

### **Post-processing**: **visual analysis of segmentations**

After obtaining and comparing the initial BOA-derived values with those of the examiners, CT images with overlaid segmentations of the aorta, PT, and autochthonous back muscles were randomly reviewed to identify potential confounding factors. This post hoc analysis was conducted using in-house datasets for each of the segmentation modifications described below. ITK-SNAP 4.0.2 (University of Pennsylvania) was used for image visualization. For each modification, a box plot of HU within the segmented voxels was generated to visualize changes in image parameters relevant to CNR calculation.

### **Post-processing**: **segmentation modifications**

Following the initial analysis, we implemented a series of modifications to the BOA-derived segmentations and repeated the CNR calculations accordingly:

1. Vessels: for the aorta and PT, binary erosion was applied using morphological structuring elements with kernel sizes ranging from 2 to 10 voxels. Prior to binary erosion, all CT volumes were resampled to a uniform voxel spacing to ensure a consistent physical erosion depth across examinations. The original auto-segmentations of the aorta and PT are referred to as *a_original* and *p_original*, respectively. Segmentations after erosion are denoted as *a_erode2–10* and *p_erode2–10*, with the numerical suffix indicating the kernel size.
2. Autochthonous back muscles: For the autochthonous back muscles, voxels with HU values between –200 and –40 (representing adipose tissue [22–24]) were first subtracted, followed by binary erosion with kernel sizes from 2 to 10 voxels. The original BOA muscle segmentation (m_original) includes both muscle tissue and intramuscular adipose components; fat subtraction was therefore applied as a post-processing step by removing all voxels within the predefined HU interval (–200 to –40 HU) prior to binary erosion. The unmodified segmentations are referred to as *m_original*, those after fat subtraction as *m_minus_fat*, and those after both fat subtraction and erosion as *m_erode2–10*, with the suffix again indicating the kernel size.

The results from these iterations were used to recalculate CNRs, which were then compared with one another and with the values obtained by the examiners.

### External validation

After identifying the preferred variant of the modified BOA, we performed external validation using data from The Cancer Imaging Archive (TCIA). In total, 1,308 cases from the publicly available LIDC-IDRI dataset were analyzed using the BOA Contrast Phase Recognition tool [25, 26]. A total of 179 CTA and 195 CTPA scans were identified. From these, two random subsets of 50 cases each were selected, one for CTA and one for CTPA, and independently reviewed by the examiners. The CNR values derived from the examiners’ assessments were then compared with those obtained using the selected BOA variant, following the same methodology as in the internal validation.

## Results

### Comparison of examiners

No significant differences in CNR measurements were observed among the three examiners, except for CNR_aorta_ in CTA between Examiner 1 and Examiner 3 (*p* = 0.04). Variability across readers was modest, with mean differences from 0.91 to 1.86. Average examiner CNRs were 9.49 ± 2.85 for CNR_aorta_ in CTA, 6.62 ± 3.21 in CTPA, 10.51 ± 3.31 for CNR_PT_ in CTA, and 13.87 ± 7.01 in CTPA (Table 1).

**Table 1:** Comparison of examiners

		CNR_aorta_		CNR_PT_	
		CTA	CTPA	CTA	CTPA
Examiners' mean CNR	Mean	9.49	6.62	10.51	13.87
	SD	2.85	3.21	3.31	7.01
Examiner 1 vs Examiner 2	*p*	0.48	0.81	> 0.99	> 0.99
	Mean difference	2.20	1.56	2.53	2.75
Examiner 1 vs Examiner 3	*p*	**0.04**	0.58	0.88	0.81
	Mean difference	2.18	1.73	2.49	3.96
Examiner 2 vs Examiner 3	*p*	0.81	> 0.99	> 0.99	> 0.99
	Mean difference	2.41	1.54	2.72	3.07
Examiner 1 vs examiners‘ mean	*p*	0.85	> 0.99	> 0.99	> 0.99
	Mean difference	1.23	0.99	1.52	2.01
Examiner 2 vs examiners‘ mean	*p*	> 0.99	> 0.99	> 0.99	> 0.99
	Mean difference	1.47	0.80	1.60	1.47
Examiner 3 vs examiners‘ mean	*p*	0.45	> 0.99	> 0.99	> 0.99
	Mean difference	1.37	0.94	1.49	2.10
	Mean of the mean differences	1.36	0.91	1.54	1.86

Bold values indicate statistical significance (*p* < 0.05)

### Examiners vs original BOA

CNRs from the unmodified BOA differed significantly from manual measurements for both vascular structures in CTA and CTPA (all *p* < 0.01), with mean differences up to 6.30. The underlying HU and SD values also showed markedly larger deviations for BOA compared to inter-examiner variability, for example, 21.88 HU vs 11.18 HU for PT in CTA, and 39.73 HU vs 25.25 HU for muscle SD.

### Visual analysis and systematic deviations

Inspection of BOA segmentations showed three main sources of deviation:

1. Inclusion of vessel walls and adjacent fat in vascular masks
2. Inclusion of paraspinal fat in muscle masks
3. Partial volume effects at muscle boundaries.

**Fig. 1:**
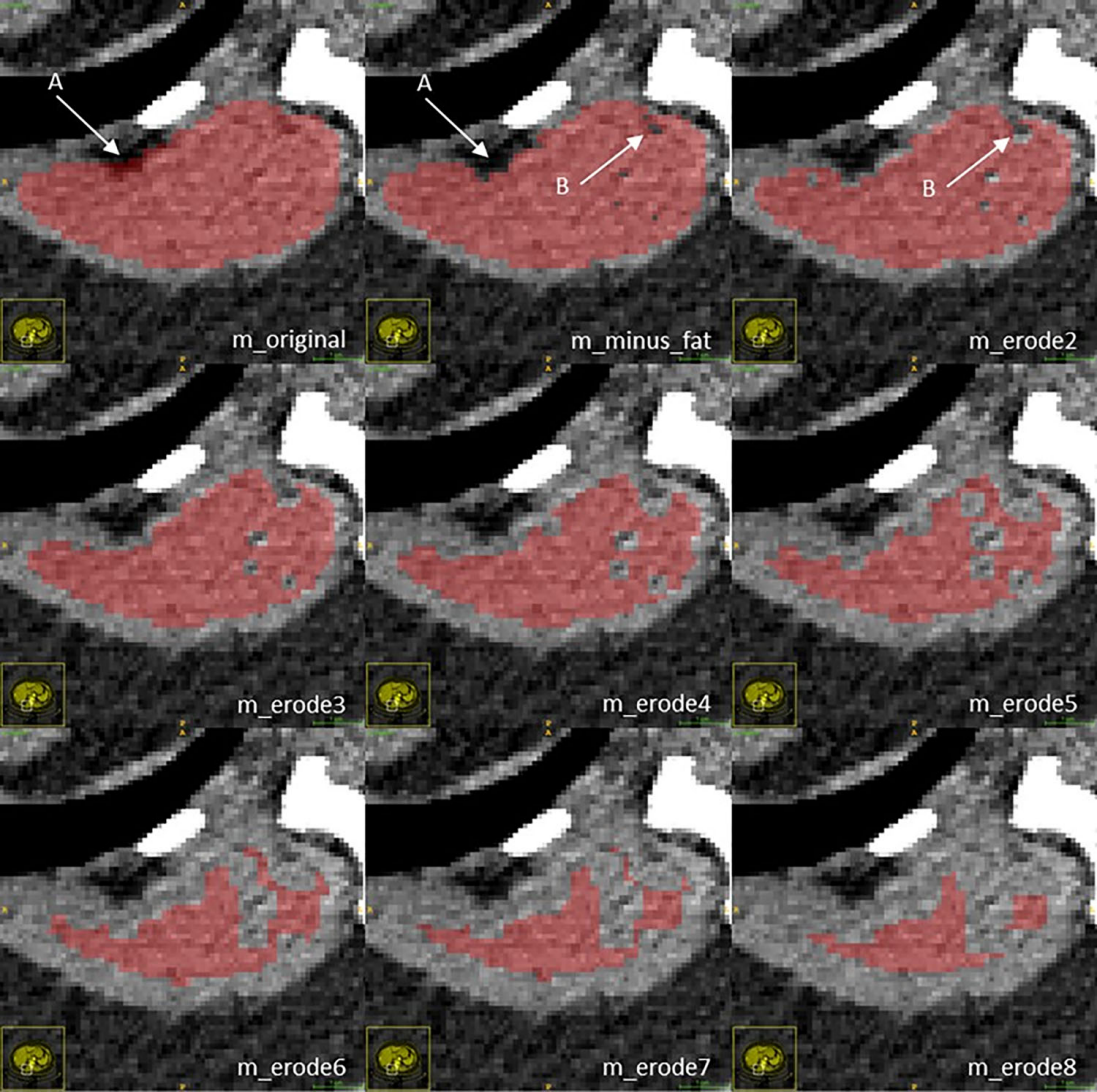
Exemplary case of the segmentation of (right-sided) autochthonous back muscles of the original and modified BOA. This illustrates, using an exemplary case, the confounding factors identified in the segmentation process and how these were mitigated in the iterations of the modified BOA: arrow A in m_original points to an area of adjacent fat that was erroneously segmented, subtracted in m_minus_fat. In m_minus_fat, a fringe-like partial volume effect between the subtracted fat and the muscle is still segmented and is largely removed by m_erode2. Arrow B in m_minus_fat indicates individual subtracted fat-dense voxels, part of a delicate fat layer. In m_erode2-8, an erosion of the segmentation around these voxels is observed, removing adjacent hypodense but non-fat-dense voxels caused by a partial volume effect from the delicate intersecting fat layer

**Fig. 2:**
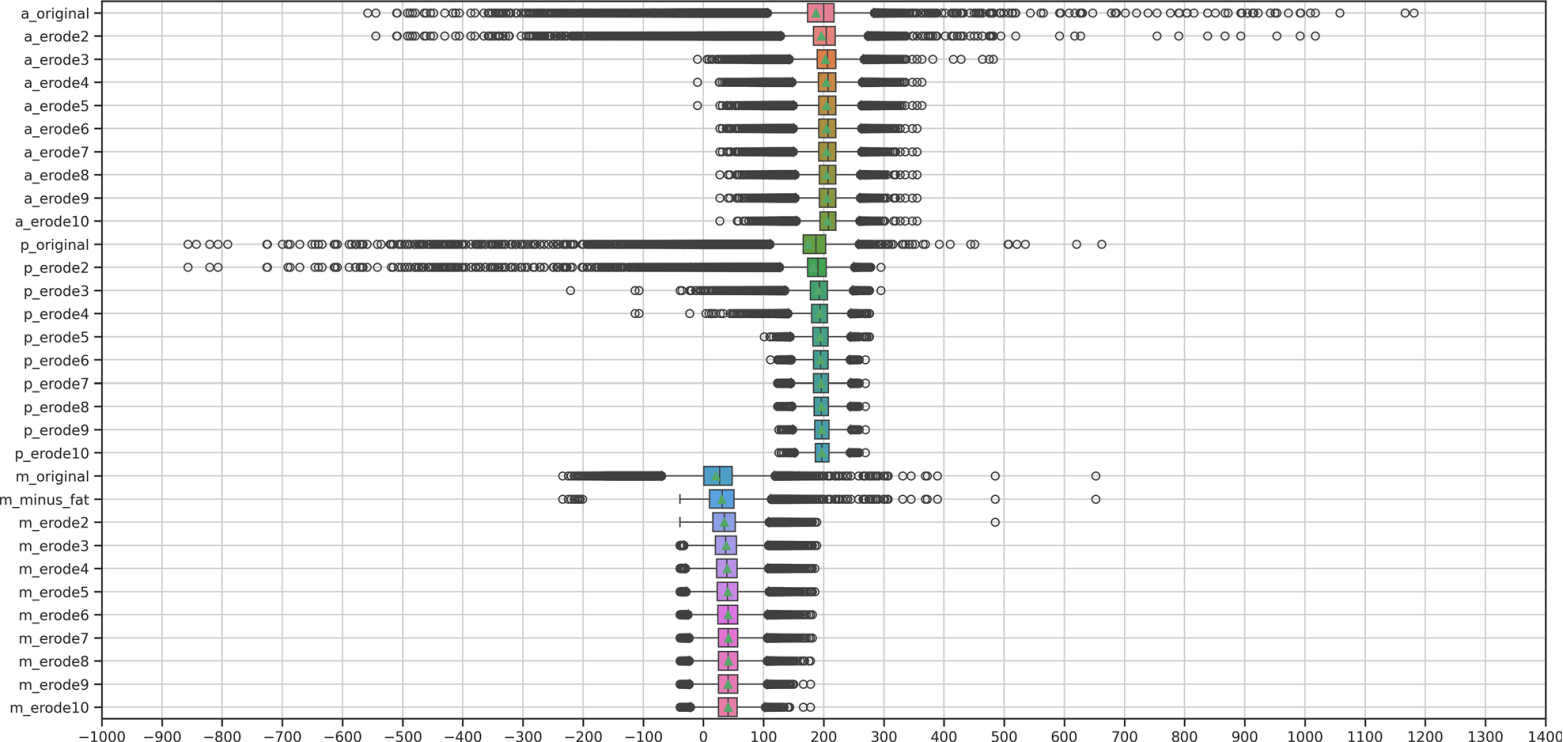
Exemplary case of voxel distribution on a HU scale within the segmentations of the original and modified BOA. The respective segmented voxels on a HU scale are plotted, illustrating, based on a sample case (the same case as in Fig. 1), parameter changes for CNR calculation throughout the modifications of BOA

These effects decreased with fat subtraction and progressive binary erosion (Fig. 1), as reflected by increasing vascular/muscle HU and decreasing muscle SD (Fig. 2).

### **Modified BOA performance**

**Fig. 3:**
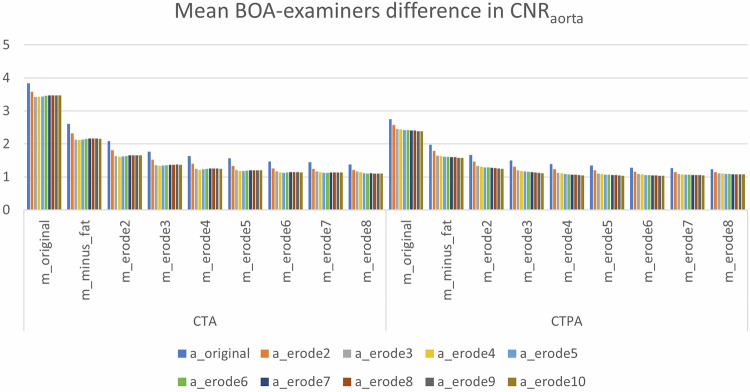
Mean BOA-examiners difference in CNR_aorta_

Modifications yielded stepwise improvements. For the aorta, mean BOA-examiners differences decreased from 3.84 (original) to 1.38 with m_erode8 (Supplementary Table 1 and Fig. 3).

**Fig. 4:**
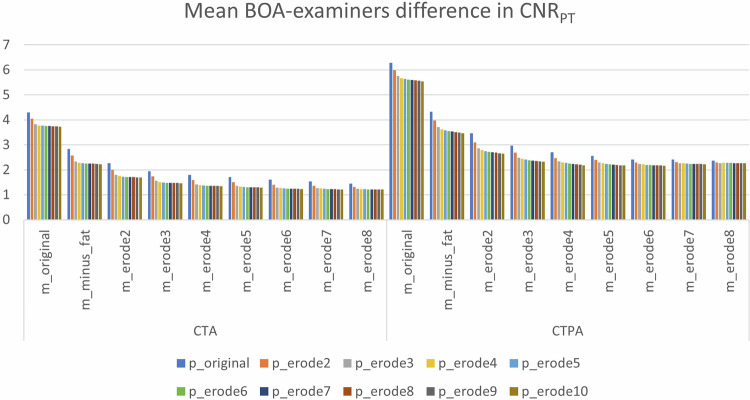
Mean BOA-examiners difference in CNR_PT_

Similar trends were observed in the PT, where differences dropped from 6.28 to 2.36 (Supplementary Table 2 and Fig. 4).

Statistical significance vs readers was lost once erosion depths of 4–6 were applied. Very deep erosions (> 8) occasionally produced non-analyzable segmentations (volume = 0 mL in sarcopenic cases).

### ROI sizes and segmentation volumes

Manual ROIs covered ~0.12 mL per structure, closely matching early-to-intermediate BOA erosions. In contrast, unmodified BOA masks spanned whole-structure volumes (aorta ~227 mL; PT ~ 77 mL; muscle ~493 mL), which decreased by 70–80% with progressive erosion (Table 2). The selected working point (a_erode6/p_erode6 with m_erode6) produced mean segmentation volumes of 126.60 mL (aorta), 36.99 mL (PT), and 143.04 mL (muscle).

**Table 2:** Volumes of auto-segmentations by BOA

		Original	minus_fat	erode2	erode3	erode4	erode5	erode6	erode7	erode8	erode9	erode10
Aorta	Mean volume (mL)	226.83		204.76	183.42	163.45	144.37	126.60	109.79	94.32	79.89	66.78
	%	100		90.27	80.86	72.06	63.65	55.81	48.40	41.58	35.22	29.44
	Min (mL)	85.70		74.97	63.65	53.29	43.75	35.13	27.46	20.73	15.04	10.35
PT	Mean volume (mL)	76.91		67.71	59.11	51.11	43.75	36.99	30.86	25.40	20.53	16.26
	%	100		88.05	76.86	66.45	56.88	48.10	40.13	33.02	26.70	21.14
	Min (mL)	27.60		22.94	18.70	14.89	11.56	8.70	6.29	4.35	2.86	1.74
Muscle	Mean volume (mL)	492.79	449.79	370.45	296.97	235.67	183.78	143.04	109.78	84.57		
	%	100	91.27	75.17	60.26	47.82	37.29	29.03	22.28	17.16		
	Min (mL)	128.83	61.87	29.45	13.74	6.07	2.41	0.74	0.20	0.03		

### Preferred variant of modified BOA

Although multiple variants of the modified BOA produced valid CNR measurements, our aim was to recommend a single consistent version applicable to all tested combinations (CNR_aorta_ and CNR_PT_ in CTA and CTPA) to facilitate implementation of automated CNR analysis. Based on statistical results and visual assessment, we selected a combination of *m_erode6* and *a_erode6* or *p_erode6*. Among the tested muscle variants (m_minus_fat and m_erode2–10), those centered around m_erode6 most frequently showed no significant difference from the examiners’ mean across a wide range of arterial combinations. This choice balances performance and robustness, avoiding both insufficient correction (as in lower erosion levels) and overcorrection (as in higher erosion levels), which were associated with significant deviations. *a_erode6* and *p_erode6* thus represent the most compatible arterial counterparts to *m_erode6*, providing consistent results without excessive erosion. Erosion level 6 represented the first configuration in which both vascular targets (aorta and PT) showed no significant deviation from readers across CTA and CTPA (p ≥ 0.35), while lower levels retained significant differences and higher levels increased segmentation failure rates without further accuracy gains.

### External validation

**Fig. 5:**
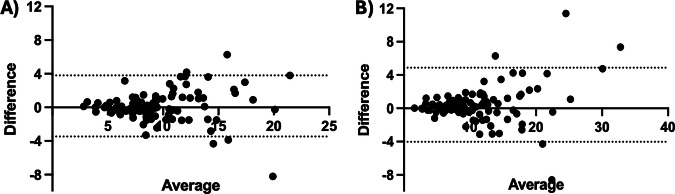
Bland–Altman analysis for external validation. Bland–Altman plots comparing CNR measurements of the preferred modified BOA variant (m_erode6 combined with a_erode6 or p_erode6) against the examiners’ mean in external validation cases from the TCIA. **A** CNR_aorta_, **B** CNR_PT_

In the TCIA cohort (*n* = 100), the preferred variant showed no significant difference from readers across all endpoints (all *p* ≥ 0.35). Mean BOA-examiners differences were 0.93 (CNR_aorta_, CTA), 1.53 (CNR_aorta_, CTPA), 0.77 (CNR_PT_, CTA), and 1.97 (CNR_PT_, CTPA); for comparison, inter-examiner differences ranged from 0.62 to 1.90. The examiners’ mean CNRs were 8.98 ± 3.18 and 10.46 ± 4.45 for CNR_aorta_ (CTA/CTPA), and 7.70 ± 3.69 and 14.74 ± 6.66 for CNR_PT_ (CTA/CTPA), respectively (Supplementary Table 3). Agreement between automated and manual CNR measurements was excellent for both vascular structures. For the aorta, the ICC indicated excellent agreement (2, 1) = 0.89, 95% CI 0.83–0.92), with a negligible bias of 0.16 and a narrow 95% LoA (–3.47 to 3.80). For the PT, agreement was similarly high (ICC (2, 1) = 0.93, 95% CI 0.90–0.95). Bland–Altman analysis revealed a slightly larger bias of 0.42 and wider LoA (–4.03 to 4.87), reflecting consistent ranking across cases despite marginally greater systematic deviation (Fig. 5).

Together, these analyses confirm strong agreement between automated and manual CNR measurements, with subtle differences in performance between vascular territories.

## Discussion

This study evaluated the feasibility and accuracy of BOA-based automated CNR analysis in chest CT by comparing BOA-derived values with expert manual measurements and quantifying the impact of segmentation modifications, with the goal of establishing a reproducible, scalable method for objective CT image-quality assessment that supports both clinical and AI-driven applications.

Initial use of the unmodified BOA produced significant disparities for CNR_aorta_ and CNR_PT_ in CTA and CTPA compared to the examiners’ results. Visual analysis, corroborated by descriptive statistics, showed systematically lower mean HU in all BOA vascular and muscle masks and a higher muscle SD, yielding lower CNRs. These discrepancies stemmed from the BOA’s deliberate whole-structure segmentation, including vessel walls and intermuscular fat, both of which typically show lower HU than contrast-enhanced lumina or pure muscle. Additional partial-volume effects with adjacent tissues further amplified the deviations.

We addressed these effects by subtracting fat from the muscle mask and applying binary erosion (kernel size 2–10) to vessel and muscle segmentations. The mean BOA-examiners differences decreased progressively and plateaued around erosion levels 4–6, after which several combinations no longer differed significantly from the examiners’ mean. We selected m_erode6 with a_erode6/p_erode6 as a balanced operating point between accuracy and over-erosion. External validation on TCIA [25] confirmed the generalizability of this variant, with non-significant differences vs readers across all endpoints.

Taken together, a minimally augmented BOA can reproduce expert-level CNR measurements in CTA and CTPA while operating fully automatically.

This supports the study's aim of establishing a scalable, objective CT image-quality assessment method. It is important to interpret this agreement considering the conceptual differences between manual and automated measurements. While human readers placed single-slice ROIs that inevitably included some intramuscular fat and partial-volume effects, the automated BOA pipeline applied volumetric segmentation with fat subtraction and erosion to mitigate such influences. These approaches are not strictly equivalent, yet the convergence of results indicates that the automated pipeline provides a systematic and reproducible proxy for expert measurements, rather than an identical replication of the manual process. Manual and automated CNR measurements are based on different sampling strategies: readers used single-slice ROIs to avoid vessel walls and partial-volume effects, while BOA derives volumetric values across entire structures. Identical numerical values are therefore not expected; the excellent agreement despite this conceptual difference supports the validity of automated volumetric CNR assessment as a reproducible proxy for manual measurements.

Whereas many prior studies in automated image quality span multiple parameters, anatomic sites, acquisition settings, or even modalities [14, 24–30], we deliberately focused on CNR, a core determinant of image quality in contrast-enhanced CT [31], and evaluated it specifically in CTA/CTPA. The need for targeted modifications underscores that automatically generated measurements should not be accepted uncritically; however, after fat subtraction and moderate erosion, BOA provides a validated function for measuring CNR_aorta_ and CNR_PT_.

The choice of reference standard warrants consideration. The examiners’ mean is based on single-slice, manually placed ROIs, whereas BOA computes CNR from volumetric segmentations across all relevant slices. A volumetric approach may better reflect whole-structure signal and noise, yet the readers’ mean remains a pragmatic clinical reference; using it, we demonstrated agreement after modification. Future work should reconcile these paradigms by deriving multi-slice or 3D manual ROIs to establish a volumetric ground truth and by quantifying how slice selection influences CNR.

By automating the labor-intensive workflow of manual CNR measurement, the modified BOA enables high-throughput image-quality assessment, reduces reader workload, and allows analysis of larger datasets in shorter timeframes. This is particularly useful for protocol-optimization studies that aim to minimize radiation or contrast dose while maintaining diagnostic quality (e.g., automatic exposure control [32–34] or contrast media reduction [35–38]), for the evaluation of emerging acquisition technologies (dual-source and photon-counting CT [39–41]), and for benchmarking novel reconstruction/processing methods (iterative reconstruction [33, 42], deep learning reconstruction [36, 43]). It also facilitates large-scale investigation into relationships between body composition, image quality, and clinical outcomes [44, 45].

From an AI perspective, the approach yields consistent, volumetric CNR labels that can serve as reliable targets or covariates for dataset curation, model conditioning, and performance auditing, aligning with the growing use of AI for CT image-quality assessment [46].

Leveraging an open-source platform (BOA) promotes transparency and reproducibility; the presented modifications can be inspected, version-controlled, and extended across sites, supporting broad adoption by the research community [19].

From a practical perspective, the proposed modifications are easy to implement, can be integrated into BOA for automated use, and maintain reproducibility and openness within existing workflows. Strengths include a multi-scanner cohort, analysis of two contrast-enhanced thoracic protocols, systematic ablation of segmentation modifications, and external validation.

Limitations are the focus on CNR_aorta_ and CNR_PT_ in CTA/CTPA (other regions/phases remain to be tested); the use of single-slice ROIs as the reference vs BOA’s volumetric masks; the possibility that fixed morphological parameters under- or over-erode in edge cases (e.g., severe sarcopenia); and potential variation with reconstruction kernels or vendors not represented here.

The internal dataset included only Siemens scans with soft kernels, which may limit generalizability, although external validation using TCIA cases confirmed robustness. However, in the external validation using TCIA cases, our approach remained robust. Moreover, this work represents a proof-of-concept evaluation with a limited sample size, and larger multi-center cohorts are warranted to consolidate these findings. Future work should evaluate adaptive morphology, robust 3D noise modeling, applications in photon-counting CT, and prospective tests of whether BOA-guided quality feedback improves diagnostic performance or AI model generalization. Finally, our study focused exclusively on CNR as a surrogate for image quality. Although widely used, CNR does not reflect complementary aspects such as spatial resolution, detectability, or task-specific performance, which should be addressed in future research.

## Conclusion

A modified BOA, combining fat subtraction in the autochthonous back muscles with moderate binary erosion of vessel and muscle masks, enables fully automated, volumetric CNR computation in chest CTA/CTPA, yielding values that agree with expert readers’ measurements and generalize across external datasets. It provides a reproducible, scalable alternative to manual single-slice, ROI-based CT image-quality assessment, reduces reader workload, and generates standardized volumetric CNR metrics that support protocol optimization and AI-driven applications. This study deliberately focuses on CNR as a central quantitative parameter of contrast-enhanced CT image quality; additional image quality dimensions should be addressed in future investigations.

## Supplementary information

ELECTRONIC SUPPLEMENTARY MATERIAL

## Data Availability

The datasets generated and/or analyzed during the current study are not publicly available due to data protection regulations, but are available from the corresponding author upon reasonable request.

## Abbreviations

a_original: Auto-segmentation of aorta without modifications
a_erode2–10: Auto-segmentation of aorta and binary erosion with a kernel of 2–10
BOA: Body and organ analysis
CNR: Contrast-to-noise ratio
CT: Computed tomography
CTA: Computed tomography angiography
CTPA: Computed tomography pulmonary angiography
HU: Hounsfield units
m_original: Auto-segmentation of autochthonous back muscles without modifications
m_erode2–10: Auto-segmentation of autochthonous back muscles including fat subtraction and binary erosion with a kernel of 2–10
m_minus_fat: Auto-segmentation of autochthonous back muscles with fat subtraction and without binary erosion
p_original: Auto-segmentation of pulmonary trunk without modifications
p_erode2–10: Auto-segmentation of pulmonary trunk and binary erosion with a kernel of 2–10
PT: Pulmonary trunk
ROI: Region-of-interest
SD: Standard deviation

## Code availability

Automated processing is based on the open-source BOA framework, version v0.1.4: https://github.com/UMEssen/Body-and-Organ-Analysis/tree/v0.1.4. No proprietary code was used.
