# Supplementary material for: Automated contrast-to-noise ratio analysis in chest CT: validation of an open-source segmentation approach
Source: Insights Imaging. 2026 Apr 7;17:88. doi: 10.1186/s13244-026-02263-y (PMC13057051; doi:10.1186/s13244-026-02263-y)
Supplement: Supplementary file 1 — ELECTRONIC SUPPLEMENTARY MATERIAL [file 13244_2026_2263_MOESM1_ESM.pdf]

Automated Contrast-to-Noise Ratio Analysis in Chest CT: Validation of an Open-Source Segmentation Approach

ELECTRONIC SUPPLEMENTARY MATERIAL

Supplementary Table 1: *Comparison of examiners and modified BOA for* CNR_{aorta}

Aorta

			a_original	a_erode2	a_erode3	a_erode4	a_erode5	a_erode6	a_erode7	a_erode8	a_erode9	a_erode10
CTA	m_original	Δ	3.84	3.59	3.42	3.42	3.44	3.46	3.47	3.47	3.47	3.47
		**p**	**< 0.01**	**< 0.01**	**< 0.01**	**< 0.01**	**< 0.01**	**< 0.01**	**< 0.01**	**< 0.01**	**< 0.01**	**< 0.01**
	m_minus_fat	Δ	2.61	2.33	2.13	2.12	2.14	2.15	2.16	2.16	2.16	2.16
		**p**	**< 0.01**	**< 0.01**	**< 0.01**	**< 0.01**	**< 0.01**	**< 0.01**	**< 0.01**	**< 0.01**	**< 0.01**	**< 0.01**
	m_erode2	Δ	2.08	1.81	1.63	1.61	1.63	1.64	1.65	1.66	1.66	1.65
		**p**	**< 0.01**	**< 0.01**	**< 0.01**	**< 0.01**	**< 0.01**	**< 0.01**	**< 0.01**	**< 0.01**	**< 0.01**	**< 0.01**
	m_erode3	Δ	1.77	1.53	1.35	1.33	1.34	1.36	1.37	1.37	1.37	1.37
		**p**	**< 0.01**	**< 0.01**	**< 0.01**	**< 0.01**	**< 0.01**	**< 0.01**	**< 0.01**	**< 0.01**	**< 0.01**	**< 0.01**
	m_erode4	Δ	1.63	1.40	1.25	1.22	1.23	1.24	1.26	1.26	1.26	1.25
		**p**	**< 0.01**	**< 0.01**	0.39	0.89	0.81	0.32	0.19	0.16	0.30	0.59
	m_erode5	Δ	1.56	1.33	1.21	1.18	1.18	1.19	1.21	1.21	1.21	1.20
		**p**	**< 0.01**	**< 0.01**	> 0.99	> 0.99	> 0.99	> 0.99	> 0.99	> 0.99	> 0.99	> 0.99
	m_erode6	Δ	1.47	1.26	1.17	1.14	1.13	1.13	1.15	1.14	1.14	1.14
		**p**	**< 0.01**	0.05	> 0.99	> 0.99	> 0.99	> 0.99	> 0.99	> 0.99	> 0.99	> 0.99
	m_erode7	Δ	1.44	1.25	1.17	1.15	1.12	1.12	1.14	1.13	1.14	1.13
		**p**	**< 0.01**	0.55	> 0.99	> 0.99	> 0.99	> 0.99	> 0.99	> 0.99	> 0.99	> 0.99
	m_erode8	Δ	1.38	1.22	1.17	1.15	1.11	1.10	1.11	1.11	1.11	1.10
		**p**	**< 0.01**	> 0.99	> 0.99	0.18	0.08	0.29	0.47	0.63	0.43	0.23
CTPA	m_original	Δ	2.75	2.58	2.46	2.44	2.42	2.42	2.41	2.41	2.39	2.38
		**p**	**< 0.01**	**< 0.01**	**< 0.01**	**< 0.01**	**< 0.01**	**< 0.01**	**< 0.01**	**< 0.01**	**< 0.01**	**< 0.01**
	m_minus_fat	Δ	1.97	1.78	1.65	1.63	1.61	1.61	1.60	1.60	1.58	1.57
		**p**	**< 0.01**	**< 0.01**	**< 0.01**	**< 0.01**	**< 0.01**	**< 0.01**	**< 0.01**	**< 0.01**	**< 0.01**	**< 0.01**
	m_erode2	Δ	1.67	1.47	1.34	1.31	1.29	1.29	1.28	1.27	1.26	1.25
		**p**	**< 0.01**	**< 0.01**	**< 0.01**	**< 0.01**	**< 0.01**	**< 0.01**	**< 0.01**	**< 0.01**	**< 0.01**	**< 0.01**
	m_erode3	Δ	1.50	1.32	1.20	1.18	1.17	1.16	1.15	1.14	1.12	1.12

	**p**	**< 0.01**	**< 0.01**	**< 0.01**	**< 0.01**	**< 0.01**	**< 0.01**	**< 0.01**	**< 0.01**	**< 0.01**	**0.01**
m_erode4	Δ	1.39	1.23	1.13	1.11	1.09	1.08	1.07	1.07	1.06	1.05
	**p**	**< 0.01**	**< 0.01**	**0.03**	**0.03**	0.11	**0.03**	0.15	0.14	> 0.99	> 0.99
m_erode5	Δ	1.34	1.20	1.11	1.09	1.07	1.07	1.06	1.06	1.05	1.04
	**p**	**< 0.01**	**< 0.01**	0.36	0.46	> 0.99	0.65	> 0.99	> 0.99	> 0.99	> 0.99
m_erode6	Δ	1.28	1.16	1.09	1.08	1.06	1.06	1.05	1.05	1.04	1.04
	**p**	**< 0.01**	**< 0.01**	> 0.99	> 0.99	> 0.99	> 0.99	> 0.99	> 0.99	> 0.99	> 0.99
m_erode7	Δ	1.27	1.15	1.09	1.08	1.07	1.07	1.06	1.06	1.05	1.05
	**p**	**< 0.01**	**0.04**	> 0.99	> 0.99	> 0.99	> 0.99	> 0.99	> 0.99	> 0.99	> 0.99
m_erode8	Δ	1.24	1.15	1.11	1.10	1.09	1.09	1.08	1.08	1.08	1.08
	**p**	**< 0.01**	> 0.99	> 0.99	> 0.99	> 0.99	> 0.99	> 0.99	> 0.99	> 0.99	> 0.99

Δ = mean BOA-examiners difference

Bold values indicate statistical significance (p < 0.05)

Supplementary Table 2: *Comparison of examiners and modified BOA for CNR_{PT}*

Pulmonary Trunk

		p_original	p_erode2	p_erode3	p_erode4	p_erode5	p_erode6	p_erode7	p_erode8	p_erode9	p_erode10
m_original	Δ	4.29	4.05	3.84	3.78	3.77	3.75	3.76	3.75	3.75	3.73
	**p**	**< 0.01**	**< 0.01**	**< 0.01**	**< 0.01**	**< 0.01**	**< 0.01**	**< 0.01**	**< 0.01**	**< 0.01**	**< 0.01**
m_minus_fat	Δ	2.84	2.57	2.34	2.28	2.27	2.25	2.25	2.24	2.24	2.23
	**p**	**< 0.01**	**< 0.01**	**< 0.01**	**< 0.01**	**< 0.01**	**< 0.01**	**< 0.01**	**< 0.01**	**< 0.01**	**< 0.01**
m_erode2	Δ	2.27	2.01	1.81	1.75	1.73	1.72	1.72	1.71	1.70	1.69
	**p**	**< 0.01**	**< 0.01**	**< 0.01**	**< 0.01**	**< 0.01**	**< 0.01**	**< 0.01**	**< 0.01**	**< 0.01**	**< 0.01**
m_erode3	Δ	1.95	1.74	1.56	1.51	1.49	1.48	1.48	1.47	1.47	1.46
	**p**	**< 0.01**	**< 0.01**	**< 0.01**	**< 0.01**	**< 0.01**	**< 0.01**	**< 0.01**	**0.01**	**0.01**	**0.02**
m_erode4	Δ	1.80	1.59	1.42	1.39	1.37	1.36	1.36	1.36	1.36	1.35
	**p**	**< 0.01**	**< 0.01**	**0.02**	0.77	> 0.99	> 0.99	> 0.99	> 0.99	> 0.99	> 0.99
m_erode5	Δ	1.71	1.51	1.36	1.33	1.31	1.31	1.31	1.30	1.30	1.29
	**p**	**< 0.01**	**< 0.01**	> 0.99	> 0.99	> 0.99	> 0.99	> 0.99	> 0.99	> 0.99	> 0.99
m_erode6	Δ	1.61	1.41	1.29	1.27	1.25	1.25	1.25	1.24	1.24	1.23
	**p**	**< 0.01**	**0.01**	> 0.99	> 0.99	> 0.99	> 0.99	> 0.99	> 0.99	> 0.99	> 0.99
m_erode7	Δ	1.54	1.36	1.27	1.25	1.24	1.23	1.23	1.22	1.22	1.22
	**p**	**< 0.01**	0.13	> 0.99	> 0.99	> 0.99	> 0.99	> 0.99	> 0.99	> 0.99	0.56
m_erode8	Δ	1.45	1.32	1.25	1.24	1.22	1.22	1.22	1.21	1.21	1.21
CTA	**p**	**< 0.01**	> 0.99	> 0.99	0.50	0.11	**0.04**	**0.04**	**0.02**	**0.03**	**0.01**

	m_original	Δ	6.28	5.99	5.75	5.68	5.64	5.62	5.60	5.58	5.57	5.54
		**p**	**< 0.01**	**< 0.01**	**< 0.01**	**< 0.01**	**< 0.01**	**< 0.01**	**< 0.01**	**< 0.01**	**< 0.01**	**< 0.01**
	m_minus_fat	Δ	4.32	3.97	3.71	3.63	3.59	3.56	3.54	3.51	3.50	3.47
		**p**	**< 0.01**	**< 0.01**	**< 0.01**	**< 0.01**	**< 0.01**	**< 0.01**	**< 0.01**	**< 0.01**	**< 0.01**	**< 0.01**
	m_erode2	Δ	3.47	3.10	2.86	2.79	2.75	2.72	2.70	2.68	2.67	2.64
		**p**	**< 0.01**	**< 0.01**	**< 0.01**	**< 0.01**	**< 0.01**	**< 0.01**	**< 0.01**	**< 0.01**	**< 0.01**	**< 0.01**
	m_erode3	Δ	2.97	2.69	2.49	2.44	2.41	2.39	2.38	2.36	2.34	2.32
		**p**	**< 0.01**	**< 0.01**	**< 0.01**	**< 0.01**	**0.01**	**0.02**	**0.02**	0.05	0.08	0.17
	m_erode4	Δ	2.70	2.47	2.34	2.30	2.28	2.25	2.24	2.22	2.21	2.18
		**p**	**< 0.01**	**< 0.01**	0.21	> 0.99	> 0.99	> 0.99	> 0.99	> 0.99	> 0.99	> 0.99
	m_erode5	Δ	2.56	2.40	2.29	2.26	2.24	2.22	2.20	2.19	2.18	2.18
		**p**	**< 0.01**	**0.01**	> 0.99	> 0.99	> 0.99	> 0.99	> 0.99	> 0.99	> 0.99	> 0.99
	m_erode6	Δ	2.41	2.30	2.23	2.22	2.20	2.19	2.18	2.18	2.18	2.17
		**p**	**< 0.01**	> 0.99	> 0.99	> 0.99	> 0.99	> 0.99	> 0.99	> 0.99	> 0.99	> 0.99
	m_erode7	Δ	2.41	2.31	2.26	2.26	2.25	2.24	2.24	2.23	2.23	2.23
		**p**	**< 0.01**	> 0.99	> 0.99	> 0.99	> 0.99	0.83	0.62	0.29	0.17	0.08
	m_erode8	Δ	2.36	2.29	2.27	2.28	2.28	2.28	2.27	2.27	2.27	2.27
CTPA		**p**	0.24	> 0.99	> 0.99	0.12	**0.02**	**0.01**	**< 0.01**	**< 0.01**	**< 0.01**	**< 0.01**

Δ = mean BOA-examiners difference

Bold values indicate statistical significance (p < 0.05)

Supplementary Table 3: *External validation of the preferred variant of the BOA (cases from the TCIA)*

		CNR_{aorta}		CNR_{PT}	
		CTA	CTPA	CTA	CTPA
Examiners' mean	mean	8.98	10.46	7.70	14.74
	SD	3.18	4.45	3.69	6.66
Modified BOA (preferred variant) vs. examiners' mean	p	0.52	> 0.99	0.35	> 0.99
	mean BOA-examiners difference	0.93	1.53	0.77	1.97
Examiner 1 vs. examiners' mean	p	> 0.99	> 0.99	0.11	> 0.99
	mean examiner-examiners difference	0.62	1.16	0.66	1.67
Examiner 2 vs. examiners' mean	p	> 0.99	> 0.99	0.23	0.35
	mean examiner-examiners difference	0.94	1.31	1.02	1.90
Examiner 3 vs. examiners' mean	p	> 0.99	> 0.99	0.58	> 0.99
	mean examiner-examiners difference	0.85	1.04	0.80	1.44
